# Symptom network approach for management in cancer care

**DOI:** 10.1016/j.apjon.2024.100482

**Published:** 2024-04-06

**Authors:** Minyu Liang, Siying Zhu, Wenwen Zhang, M. Tish Knobf, Zengjie Ye

**Affiliations:** School of Nursing, Guangzhou University of Chinese Medicine, Guangzhou, China; School of Nursing, Guangzhou Medical University, Guangzhou, China; Galactophore Department, Affiliated Cancer Hospital & Institute of Guangzhou Medical University, Guangzhou, China; School of Nursing, Yale University, Orange, CT, United States; School of Nursing, Guangzhou Medical University, Guangzhou, China

Oncology patients diagnosed with breast, gynecological, gastrointestinal, and lung cancer often experience ≥ 10 symptoms associated with the disease or treatment.^1^^,^^2^ Even though an individual symptom may account for a significant amount of distress, the occurrence of multiple symptoms often results in a high symptom burden, significant impairment in functioning, and poorer quality of life.^1^ There is an urgent need to identify the underlying mechanisms for symptoms in order to design effective symptom management.

## Symptom clusters

The interaction of symptoms is a sophisticated and dynamic relationship. A symptom cluster reflects two or more symptoms with specific underlying concepts that occur together and are associated with each other. The relationship among symptoms may vary over the period of the disease related to stage or progress of the disease and the treatment. Detecting symptom clusters analytically has contributed to classifying symptoms with similar mechanisms. However, only combining individual symptom interventions together without specific emphasis will result in suboptimal outcome such as marginal overall symptom improvement, resource wastage, and increased healthcare-provider costs.^3^ In other words, symptom clusters are useful for clinical practice, but the common analytical approach (i.e., K-means) could not adequately address the strength of inter-relation among symptoms within the cluster. In addition, based on the theory of unpleasant symptoms developed and updated by Lenz et al., physiologic, psychologic, and situational factors may also impact symptoms and affect function and quality of life.^4^ Thus, the search for explanations of symptom mechanisms should include the interaction of symptoms and potential effect of external environment.

## Symptom network analysis and management

Symptom network analysis represents an analytical paradigm to interpret the underlying mechanisms of symptoms from the perspective of symptom interaction as well as the effect of external environment by using associative network and causal connections. Associative network shows a nontrivial topology, in which certain symptoms are more tightly connected than others. It also identifies how different individual symptoms and the various symptom clusters are interconnected. In contrast, causal connections present as a pattern of direct causal connections between symptoms, which may also reflect how these networks change over time. This paradigm may provide important implications for precise and effective symptom management by intervening on symptoms (modifying the state of one or more symptoms based on associative network), intervening in the network (modifying the connections among network nodes based on symptom–symptom causal connections), and intervening in the external environment (eradicating or modifying the external factors).

The core symptoms and bridge symptoms identifying through symptom network analysis can be considered as critical breaking points of maximally deactivating symptoms interaction, which are the best targets of “intervening in symptom.” The core symptoms present the strongest associations with the other symptoms and play an important role in activating multiple co-occurring symptoms in global network, whereas bridge symptoms present the interaction among various symptom clusters. Thus, targeting core symptoms and bridge symptoms may maximally reduce a chain reaction among related symptoms in the overall structural networks as well as across various symptom cluster.

Symptom–symptom causal connections may greatly affect the network structure itself, which may be the optimal entry points of “intervening in the network.” Based on interventionist theories of causation, if an (experimental or natural) intervention changes the state of one symptom, this would change the probability distribution of the other symptoms.^5^ Causal connections between symptoms may be attributabe to basic biological psychological processes or homeostatic couplings, which are evolving over time and are agnostic by network theory. Thus, changing symptom–symptom causal connections generally alter the overall network structure such as changes in the network density or connectivity. For example, a positive effective intervention in the network (i.e., modifying symptom–symptom causal connections) may decrease their connectivity and reduce a cascade or chain reaction of symptom effects, leading to a decline in generally collective effect of symptoms. However, few studies explore symptom–symptom causal connections in network structure for oncology patients. In addition, most studies use lateral data, which cannot map a symptom trajectory over time. Introducing a temporal dimension to network analysis that takes us beyond cross-sectional data, but longitudinal time series data, may move us one step closer to discerning cause and more precise interventions.

Severity network models can also identify the potential effect of the external environment (i.e., sociodemographic factors, disease factors, psychological factors). According to this model, the connectivity among symptoms increases as the “burden” or the impact of the external environment grows (i.e., daily stress, traumatic experiences, etc.). The connectivity between symptoms may also be reduced if the “burden” of environmental stress diminishes. Thus, intervening in the external environment can change the levels of connectivity among network symptoms. An increasing number of studies are focusing on the influence of various demographic (i.e., age, gender) and clinical (i.e., comorbid conditions, functional status) characteristics on the network structure of cancer symptoms. Most studies have paid more attention to the influence of a single external factor, with less attention paid to the comprehensive effects of multiple external factors on the network structure. Considering the integration of multiple external factors and reducing bias selection of variables may be an important next step.

## Challenges and implications

Although symptom network analysis is promising in guiding precise and effective symptom management, it is still necessary to overcome some challenges to provide in-depth insights into the complex and dynamic relationship of symptoms. To date, the majority of studies have identified conditionally dependent relationships of symptoms within a single dimension statically as most of them used a pairwise Markov Random Field and Bayesian network in analysis of symptom relationship for oncological patients. A pairwise Markov Random Field is an undirected graphical model of a set of random variables having a Markov property, which is described associative network relationship of symptoms, whereas Bayesian network allows complex causal relationships to be represented as a directed acyclic graph with Bayesian probabilities, which show causal connections relationship of symptoms. Within these models, symptom relationships are under the hypothesis that each pair of symptoms has conditionally dependent relationships and is always used to describe the symptom relationship for just one space-time point, increasing the likelihood of unreliability in estimates of network edges and node centrality, particularly when the symptoms are substantially correlated. Thus, these models cannot explain the complex paths of dependently mutidimensional and time-resolved symptoms. Therefore, a more complex network analysis should be needed to interpretate intercorrelated, mutidimensional, and time-resolved symptoms in oncology patients. For example, the higher-order network models may have improved explanatory power for illustrating the effects of non-Markovian paths in various types of higher-order-dependent symptoms. This makes it possible to analyse the complex interplay between time and topology in networked systems and explain why non-Markovian characteristics of paths can both decelerate and accelerate dynamical processes and collective dynamics. Moreover, in this model, interdependency of mutidimensional symptoms can be addressed with multilayer graphs, resulting in the interdisciplinary analysis of complex multilayer networks and accounting for relationships among multiple layers of connectivity. This model can yield more stable and consistent outcomes of symptom community detection, symptoms ranking, and modeling dynamical processes. However, the higher-order network models in exploring the relationship of symptoms are still in their infancy and facing some challenge. It highlights the need for computational and statistical methods that use variable-order or multiorder models and model order reduction techniques to generate computationally tractable models that neither underfit nor overfit the data, which requires interdisciplinary exchanges between physics, computer science, and statistics.

## Conclusions

In conclusion, symptom network analysis is “state-of-the-art” in interpretating the underlying mechanisms of symptoms, which may provide clues to the precise and effective symptom management for oncology patients. In addition, the higher-order network models have a greater advantage to unravel complex and dynamic relationship of symptoms than the standard network models such as pairwise Markov Random Field and Bayesian network.

## Ethics statement

Not required.

## Funding

This research was funded by grants from the 10.13039/501100001809National Natural Science Foundation of China (Grant Nos. 72274043, 71904033), the Young Elite Scientists Sponsorship Program by CACM (Grant No. 2021-QNRC2-B08), the Special Fund for the Cultivation of Guangdong College Students’ Scientific and Technological Innovation (Grant No. pdjh2022b0124), and the 10.13039/501100012151Sanming Project of Medicine in Shenzhen (Grant No. SZZYSM202206014).

## CRediT authorship contribution statement

**Minyu Liang**: Wiriting original draft; **Siying Zhu and Wenwen Zhang**: Literature review; **M. Tish Knobf and Zengjie Ye**: Supervision and editing. All authors were granted complete access to all the data in the study, with the corresponding author bearing the final responsibility for the decision to submit for publication. The corresponding authors affirm that all listed authors fulfill the authorship criteria and that no others meeting the criteria have been omitted.

## Declaration of competing interest

The authors declare no conflict of interest. The corresponding author, Professor Zengjie Ye, serves as a member of the editorial board of the *Asia-Pacific Journal of Oncology Nursing*. The article has undergone the journal's standard publication procedures.

## Data availability statement

Data availability is not applicable to this article as no new data were created or analyzed in this study.

## Declaration of Generative AI and AI-assisted technologies in the writing process

No AI tools/services were used during the preparation of this work.
